# Tumor associated macrophages in gastric cancer dual roles in immune evasion and clinical implications for targeted therapy

**DOI:** 10.3389/fimmu.2025.1706744

**Published:** 2025-12-11

**Authors:** Chang Wang, Xu Fan, Xiaomen Sun, Yi Xu, Yanna Sun, Jidong Liu

**Affiliations:** 1 College of Continuing Education, Liaoning University of Traditional Chinese Medicine, Shenyang, Liaoning, China; 2 College of Acupuncture and Tuina, Liaoning University of Traditional Chinese Medicine, Shenyang, Liaoning, China; 3 Key Laboratory of The Theory and Application of Viscera in Chinese Medicine, Ministry of Education, Liaoning University of Traditional Chinese Medicine, Shenyang, Liaoning, China

**Keywords:** gastric cancer, tumor-associated macrophages, tumor microenvironment, immune evasion, prognosis, immunotherapies

## Abstract

Gastric cancer (GC) remains a major global health burden with persistently high mortality despite therapeutic advances. Accumulating evidence highlights the pivotal role of tumor-associated macrophages (TAMs) in orchestrating gastric tumor progression through immune suppression, angiogenesis, extracellular matrix remodeling, and metastasis. Within the tumor microenvironment (TME), TAMs exhibit functional plasticity, often polarizing toward an M2-like phenotype that promotes immunosuppression and tumorigenicity. These cells actively participate in immune evasion via immune checkpoint expression and cytokine-mediated T cell inhibition, while also facilitating lymphovascular invasion and chemoresistance through exosome-mediated crosstalk. The density and phenotype of TAMs have been associated with prognosis and therapeutic response in GC. Recent studies have proposed TAMs as promising targets for therapy, with strategies focusing on depleting M2 subsets, reprogramming toward M1 phenotypes, and blocking TAM-driven oncogenic signaling. Targeted interventions, including MENK, paclitaxel, and NF-κB inhibitors, have shown potential in preclinical models. This review comprehensively discusses the mechanistic roles of TAMs in GC and evaluates emerging TAM-targeted therapeutic strategies that may enhance the efficacy of immunotherapy and improve patient outcomes.

## 1 Introduction

Gastric cancer (GC) remains one of the most lethal malignancies worldwide, ranking fourth in cancer-related mortality following lung and liver cancers (1, 2). Despite substantial advances in diagnostic and therapeutic strategies over recent decades, the global incidence and mortality of GC have exhibited only marginal improvement (1, 3). The pathogenesis of GC represents a protracted, multifactorial process involving a dynamic interplay between genetic, epigenetic, environmental, and immunological determinants. Chronic gastric inflammation, particularly that induced by persistent *Helicobacter pylori* infection, has long been recognized as a pivotal initiator of the neoplastic cascade within the stomach (4, 5). More recently, attention has shifted toward the immunological landscape of GC, where the tumor immune microenvironment has emerged as a central orchestrator of tumor initiation, progression, and therapeutic response (6–8).

The tumor microenvironment (TME) has emerged as a pivotal determinant of oncogenesis and tumor progression (9–11). Within this complex niche, tumor-associated macrophages (TAMs) have attracted considerable attention as central modulators of cancer biology. Beyond their canonical roles in innate immunity, TAMs exhibit functional plasticity, exerting both tumor-suppressive and tumor-promoting effects depending on contextual cues (12, 13). In GC, TAMs constitute a predominant immune subset that drives diverse malignant processes, including tumor cell proliferation, invasion, angiogenesis, and immune modulation (14). Importantly, higher TAM abundance has been positively linked to deeper tumor infiltration, nodal metastasis, and advanced clinical stages. A meta-analysis identified a marked reduction in overall survival among GC patients exhibiting elevated TAM infiltration, reinforcing the prognostic significance of macrophage burden in these tumors (15). Coupling TAM-associated metrics with the conventional TNM staging system could yield improved risk stratification strategies. This review synthesizes emerging insights into the functional and clinical implications of TAMs in the pathobiology of GC.

## 2 Roles of TAMs

Within the tumor microenvironment, immune and stromal components engage in reciprocal signaling that dictates cellular fate decisions and functional reprogramming (16–20). Biochemical inputs, including cytokines, growth factors, oxygen gradients, and nutrient availability, coordinately shape the transcriptional and metabolic landscapes of resident cell populations (21). TAMs, predominantly derived from monocytes recruited via chemokines like CCL2 and M-CSF, are profoundly shaped by this milieu and adopt functional phenotypes that facilitate immune evasion, angiogenesis, metastasis, and tumor cell proliferation (22). Upon entry into the tumor niche, monocytes differentiate into macrophages in response to local cues and subsequently polarize along a malleable M1–M2 spectrum (23). Classically activated M1 macrophages, induced by IFN-γ, LPS, or GM-CSF, produce IL-1β, IL-12, IL-23, and TNF-α, express inducible nitric oxide synthase (iNOS), and exhibit enhanced antigen presentation and tumoricidal capacity. In contrast, alternatively activated M2 macrophages, driven by IL-4, IL-13, or M-CSF, express CD163 and CD206, secrete IL-10, and suppress inflammation while promoting extracellular matrix (ECM) remodeling, angiogenesis, and immunosuppression (24, 25). Notably, TAMs rarely align with a strict binary classification *in vivo*, often co-expressing M1 and M2 markers in hybrid states (26). Their phenotypic heterogeneity reflects complex transcriptional, metabolic, and epigenetic reprogramming. Hypoxia stabilizes hypoxia-inducible factor-1α (HIF-1α), promoting glycolysis and VEGF expression, which drive angiogenesis and M2 polarization (27, 28). Similarly, PPARγ facilitates fatty acid metabolism and the transcription of anti-inflammatory genes, sustaining M2-like traits (29). STAT3, activated downstream of IL-6 or tumor-derived cytokines, orchestrates expression of arginase-1, IL-10, and CD163—key mediators of immunosuppressive reprogramming (30). Together, these pathways converge to enforce the metabolic and functional plasticity of TAMs in hypoxic niches of GC, reinforcing their tumor-supportive identity. This adaptability, while fostering disease progression, simultaneously renders TAMs pharmacologically targetable. Therapeutic strategies aimed at reprogramming TAMs toward an M1-like phenotype hold promise for restoring antitumor immunity and overcoming the immunosuppressive barrier characteristic of the gastric TME (31).

## 3 TAMs and immune response

TAMs are deeply involved in facilitating tumor immune evasion, particularly through their ability to establish immune tolerance during early and progressive stages of tumorigenesis (22, 32). This immunosuppressive cascade involves a complex interplay among various immune constituents, especially T lymphocytes, macrophages, and auxiliary regulatory cell types, each contributing to the modulation of immune surveillance (33). Central to this suppressive machinery is the engagement of immune checkpoint pathways. Molecules such as programmed cell death protein 1 (PD-1) and cytotoxic T-lymphocyte antigen 4 (CTLA-4), along with their respective ligands, constitute the foundation of this regulatory axis (34). These immune checkpoints, frequently upregulated on effector lymphocytes following activation, act as essential modulators to curb uncontrolled immune activation (35). Their ligands, including programmed death-ligand 1 (PD-L1), PD-L2, and members of the B7 family (CD80/CD86), bind to PD-1 or CTLA-4 and suppress antigen receptor–mediated downstream signaling in both T and B cells (36).

Interestingly, PD-L1 is abundantly expressed by inflammatory macrophages, whereas PD-L2 is largely absent under basal conditions but becomes inducible under the influence of cytokines such as IL-4 (37). This divergent expression profile points toward distinct functional specializations in the regulation of type 1 versus type 2 immune polarization (38). Meanwhile, evolving changes within the TME promote the emergence of immune editing, a process wherein malignant cells acquire the ability to circumvent immune-mediated destruction. Tumor-derived cytokines and chemokines facilitate the recruitment of immunosuppressive actors such as myeloid-derived suppressor cells (MDSCs), regulatory T cells (Tregs), and TAMs, thereby establishing an immune-permissive niche favorable to tumor progression (39). Another defining feature of TAM activity in the cancer milieu is their promotion of tumor angiogenesis. Hypoxia within tumor regions serves as a powerful stimulus for this process (40). TAMs are known to preferentially localize within oxygen-deprived zones, guided by gradients of chemoattractants like vascular endothelial growth factor (VEGF) and endothelin-2, which are markedly elevated under such conditions (28, 39). Their accumulation within hypoxic compartments is closely tied to phenotypic reprogramming toward a more pro-angiogenic and tumor-supportive state (41). This functional shift may underlie the variable clinical responses to anti-angiogenic therapies such as ramucirumab, particularly across molecularly distinct subgroups of GC.

## 4 TAMs and GC progression

In both human GC specimens and murine tumor models, TAMs predominantly assume an M2-like polarization state. In particular, peritoneal fluid samples from patients with metastatic spread to the peritoneum demonstrate a significantly elevated proportion of M2-skewed TAMs relative to non-metastatic cases (42). These macrophages exhibit suppressed antigen presentation and cytotoxicity, while simultaneously overexpressing a repertoire of pro-tumorigenic factors, including VEGF, epidermal growth factor (EGF), semaphorin 4D (Sema4D), matrix metalloproteinases (MMPs), and cathepsins, which are instrumental in fostering chromosomal instability, angiogenesis, lymphatic vessel formation, extracellular matrix remodeling, tissue invasion, and immune suppression (40). Furthermore, tumor cells actively shape the immune microenvironment by secreting immunomodulatory cytokines such as CCL2, M-CSF, TNF, IL-10, and TGF-β, thereby amplifying the recruitment and M2 polarization of TAMs (43). This cytokine-mediated feedback loop serves to reinforce immune escape mechanisms and enhance tumor progression. Importantly, the phenotypic characteristics of TAMs are not uniform; they vary considerably depending on tumor histotype, anatomical compartment, and disease stage, reflecting the plasticity and contextual responsiveness of these immune cells within the evolving tumor ecosystem.

### 4.1 TAMs in tumor angiogenesis and lymphangiogenesis

Neovascularization, encompassing both angiogenesis and lymphangiogenesis, is indispensable for continuous tumor expansion and metastatic dissemination (44). Within the GC milieu, TAMs act as potent mediators of vascular remodeling by secreting vascular endothelial growth factors, particularly VEGF-A and VEGF-C, which facilitate the formation of blood and lymphatic vessels (45, 46). This activity appears to be regulated through pathways involving NF-κB and vasohibin-1 (47). Exposure to hypoxic stress within the TME induces TAMs to upregulate hypoxia-responsive transcription factors, including HIF-1 and HIF-2. These factors, in turn, activate downstream signaling networks that drive oncogenic processes such as cell proliferation, invasion, vascular sprouting, and immune modulation (28). Hypoxic zones also enhance TAM recruitment and activate associated signaling circuits, resulting in increased secretion of VEGFs and heightened proteolytic activity via matrix metalloproteinases MMP-2 and MMP-9, thereby fostering the development of microvasculature in GC tissues (48). Furthermore, TAMs have been implicated in facilitating the dissemination of GC cells via the lymphatic system, thereby accelerating both local invasion and distant metastasis of malignant cells (49). A pivotal early event in lymph node colonization may involve crosstalk between TAMs and lymphatic endothelial cells resident within lymph nodes of GC patients, contributing to the initiation and progression of lymphangiogenic pathways (50). High-mobility group box 1 (HMGB1) is another factor correlated with nodal spread in gastric malignancies. By engaging the receptor for advanced glycation end products (RAGE), HMGB1 enhances the protumorigenic polarization of M2-like macrophages, which in turn augments the invasive capacity of co-cultured MKN-45 gastric cancer cells (51).

### 4.2 TAMs in tumor invasion and metastasis

Extensive *in vitro* and *in vivo* studies have established TAMs as pivotal drivers of GC invasion and metastasis. TAMs promote tumor progression via key signaling pathways, including TGF-β/BMP, TNF-α/Wnt, IL-6/STAT3/IRF4, and Wnt/β-catenin, each regulating cell motility, survival, and immune suppression (48, 52, 53). Notably, the TGF-β/BMP axis induces epithelial–mesenchymal transition (EMT), enhancing tumor cell invasiveness, while concurrently facilitating regulatory T cell (Treg) differentiation and inhibiting cytotoxic immunity (54, 55). In parallel, IL-6/STAT3/IRF4 signaling promotes M2-like polarization, upregulation of anti-apoptotic genes, and angiogenesis, while impairing dendritic cell (DC) maturation and augmenting immune checkpoint expression (56). Multiple mediators, including CCL5, kindlin-2, ADAM8/9, TIMP3, TIM-3, Sema4D, and osteopontin (OPN), further support GC proliferation and dissemination (48, 52, 53). HMGB1 is overexpressed in GC and closely associated with GC progress and poor prognosis (57). By binding RAGE, HMGB1 enhances M2 polarization, invasiveness of MKN-45 cells, and expression of MMP-9 and mucin-1, thereby accelerating angiogenesis (51). TAM-induced EMT is further potentiated via FOXQ1 upregulation and TGF-β activation, correlating with aggressive histopathological phenotypes (58, 59).

Exosome-mediated crosstalk between tumor cells and macrophages reinforces this pro-tumoral feedback loop. GC-derived exosomes shuttle regulatory cargo—such as miRNAs and lncRNAs—to reprogram macrophages toward an M2 phenotype (60). For instance, exosomal miR-155 and miR-21 suppress SOCS1 and PTEN, respectively, thereby driving immunosuppressive polarization and metastatic capacity (61). Reciprocally, TAM-derived exosomes potentiate tumor aggressiveness. M2-exosomes enriched with apolipoprotein E (ApoE) remodel the actin cytoskeleton and enhance GC cell migration via PI3K/AKT signaling (62). Collectively, these bidirectional exosomal circuits establish a self-sustaining immunosuppressive niche that drives metastasis and therapy resistance. Disrupting these intercellular networks may yield novel strategies to reverse immune escape and overcome chemoresistance in GC.

### 4.3 TAMs and immune evasion in GC

Immune evasion is a hallmark of GC, with TAMs playing a central role in sculpting an immunosuppressive microenvironment. Notably, TAMs in GC lesions exhibit elevated expression of the immune checkpoint receptor PD-1, a feature absent in adjacent normal tissue (63). These PD-1^+^ TAMs adopt an M2-like phenotype, directly engaging PD-L1–expressing tumor cells and secreting IL-10, thereby inhibiting CD8^+^ T cell proliferation and effector function (63, 64). Concurrently, they impair DC maturation by releasing IL-10 and TGF-β, downregulating MHC-II and co-stimulatory molecules (CD80, CD86), and blunting T cell priming. PD-1^+^ TAMs also suppress natural killer (NK) cell cytotoxicity via TGF-β–mediated repression of perforin and granzyme B, a mechanism reinforced by TAM-derived exosomal miRNAs targeting NK activation pathways. Tumor-derived exosomes further amplify immune suppression by driving circulating monocytes toward a PD-1^+^ M2-like phenotype, establishing a self-reinforcing immunoregulatory loop. TAMs also secrete TGF-β, IL-10, and arginase 1, which collectively inhibit antigen presentation, impair CD8^+^ T cell function, and promote regulatory T cell (Treg) expansion. Arginase 1 depletes extracellular arginine, a critical substrate for T cell metabolism, fostering CD8^+^ T cell exhaustion. These mechanisms underscore the rationale for dual targeting of PD-1^+^ TAMs and tumor-derived exosomes to restore immune competence in GC (65, 66). In parallel, recent evidence implicates layilin (LAYN) is over−expressed in GC, which exhibits a strong association with macrophage infiltration, particularly the M2-polarized phenotype, as well as with markers indicative of regulatory T cells and T cell exhaustion (67). Beyond immune modulation, TAM-derived exosomes contribute to chemotherapy resistance. Specifically, M2-TAMs transfer miR-21 via exosomes to GC cells, downregulating PTEN and activating the PI3K/AKT cascade, thereby inhibiting apoptosis and promoting cisplatin resistance (68). These findings highlight the multifaceted role of TAMs in immune evasion and treatment failure and suggest that disrupting TAM–exosome interactions may improve therapeutic efficacy in GC.

### 4.4 TAMs and prognostic implications in GC

Chronic inflammatory stimuli, often triggered by microbial infections or environmental insults, precede the development of many solid tumors (39, 69, 70). In GC, *Helicobacter pylori*–driven inflammation constitutes a hallmark of the TME, modulating immune cell dynamics and disease trajectory (71). This chronic inflammatory milieu suppresses M1 macrophage activation, promotes M2 polarization, elevates reactive oxygen species (ROS), and induces macrophage apoptosis, cumulatively accelerating tumor progression (41, 72, 73). M2 macrophage enrichment correlates with adverse outcomes across cancers, including GC, particularly in the context of heightened TGF-β signaling (74, 75). In metastatic GC, increased M2 density, marked by CD204 expression, predicts reduced overall survival (OS) and serves as a negative prognostic marker (14). PD-L1^+^ TAMs show impaired phagocytosis and suppress NK cell cytotoxicity via TGF-β pathways, enhancing immune evasion (76). Notably, M2 macrophages are more abundant in poorly differentiated tumors, while signet-ring and mucosal subtypes exhibit lower levels (77). Meta-analyses confirm that M2 infiltration correlates with worse prognosis, whereas M1 dominance is linked to improved outcomes (78).

**Figure 1:**
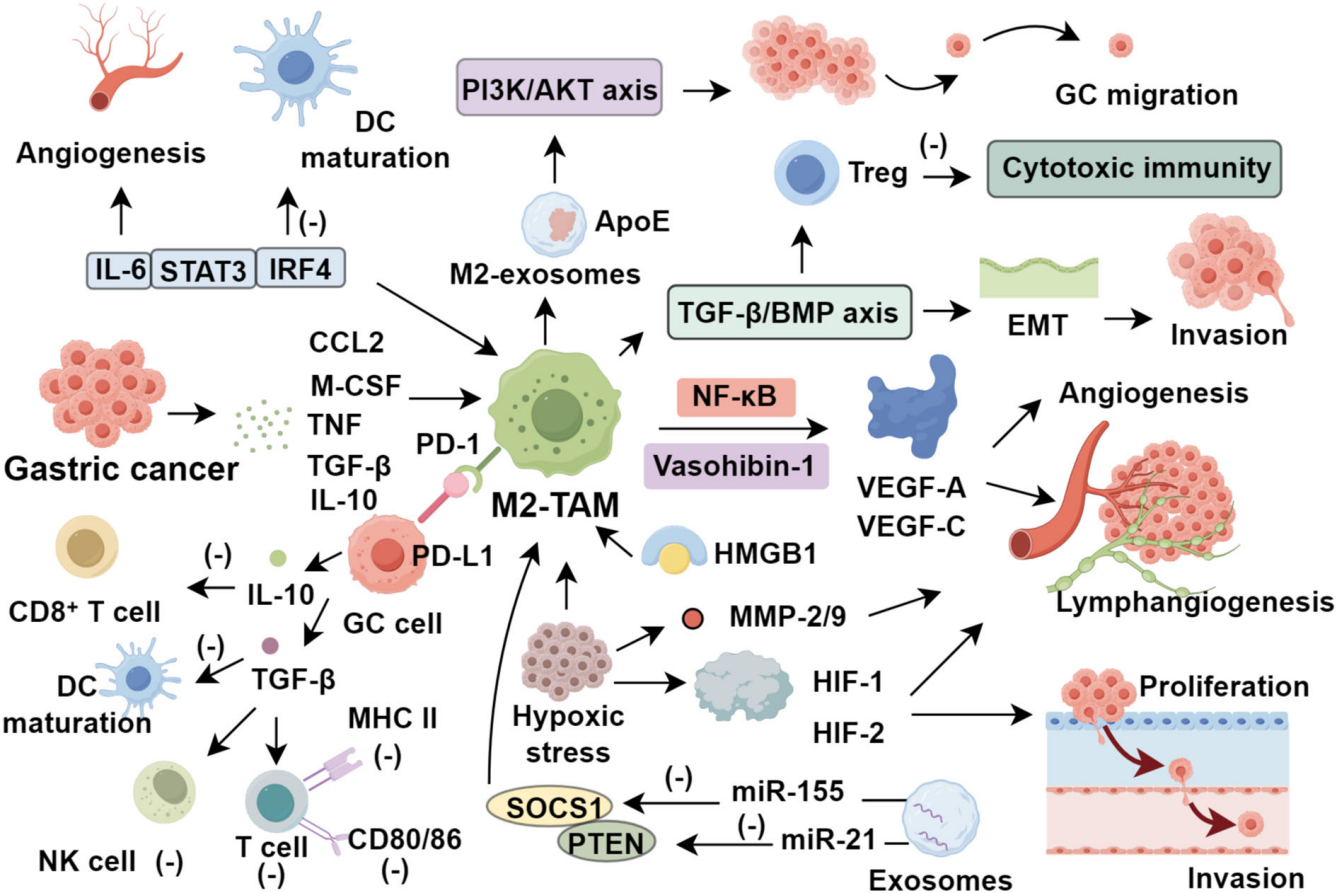
Role of tumor-associated macrophages in gastric cancer.

Molecular profiling has revealed subtype-specific TAM distributions. CD68^+^ TAMs are enriched in genomically stable (GS) and diffuse-type GC, while CD68^+^CD206^+^ M1-like TAMs are more common in microsatellite instability (MSI) and intestinal-type tumors. Although PD-L1 expression is reduced in GS and diffuse types, this does not appear prognostically relevant (79). Principal component analysis of the TME identifies three immunological phenotypes: high TME scores reflect antiviral and interferon-α signaling, while low scores indicate TGF-β dominance, hypoxia, and angiogenesis—conditions linked to T cell suppression and poor outcomes (80). Diffuse-type GC exhibits overexpression of extracellular matrix (ECM) remodeling and angiogenesis genes, inversely correlating with immune activation signatures and suggesting ECM-driven immune exclusion (81). Single-cell transcriptomics further underscores the immunological complexity of GC, revealing elevated stromal and exhausted cytotoxic T cell populations within tumors, relative to adjacent tissue. TAMs demonstrate substantial functional heterogeneity beyond the M1/M2 dichotomy and express diverse immune checkpoint molecules (82). These findings underscore the need for high-resolution immunoprofiling to delineate immunosuppressive circuits and identify precision immunotherapeutic targets within the GC TME (**Figure 1**).

## 5 Therapeutic targeting of TAMs in GC

Surgical resection, combined with adjunctive modalities such as perioperative chemotherapy, radiotherapy, and immunotherapy, continues to serve as the primary treatment paradigm for GC (83). However, increasing attention is being directed toward the non-malignant cellular constituents of the TME, particularly due to their relative genomic stability compared to neoplastic cells. This stability renders them amenable to pharmacological targeting. Within this stromal milieu, TAMs play a central role in fostering tumor progression and suppressing antitumor immunity in GC, positioning them as viable therapeutic targets (84). Interventions aimed at impairing TAM-mediated immunosuppressive signaling have shown promise in mitigating angiogenesis, lymphatic remodeling, invasion, and metastatic dissemination, while concurrently reactivating immune surveillance mechanisms (12). Recent investigations have revealed specific molecules and pharmacologic agents capable of redirecting TAM polarization toward a tumor-inhibitory M1-like phenotype by impeding tumor-supportive signaling cascades (85). Methionine enkephalin (MENK), an endogenous opioid pentapeptide originating from proenkephalin A, has demonstrated the ability to elevate opioid growth factor receptor (OGFr) expression and inhibit PI3K/AKT/mTOR signaling in both TAMs and gastric tumor cells (86). This pathway inhibition promotes macrophage repolarization from the M2 phenotype to M1, thereby suppressing cancer cell proliferation and enhancing apoptotic responses (86). Likewise, administration of low-dose paclitaxel (PTX) has been reported to stimulate the TLR4/NF-κB p65 signaling cascade, which facilitates phenotypic reprogramming of TAMs in GC tissues, leading to enhanced antitumor immunity alongside direct cytotoxic effects (87).

Beyond polarization, strategies designed to eliminate TAMs or destabilize the M2 state are also under exploration. The NF-κB signaling pathway, widely implicated in inflammation-driven malignancies, constitutes a critical node in macrophage-mediated tumor support (88). Studies in murine models of colorectal and hepatic carcinomas have confirmed the indispensable role of NF-κB activation in sustaining tumor-associated inflammatory responses (89). Members of the TNF cytokine family, particularly TNF-α, initiate transcriptional programs through NF-κB signaling that drive both inflammatory and anti-apoptotic processes. Upon stimulation by TNF-α, NF-κB translocates from the cytoplasm into the nucleus, inducing a range of genes involved in inflammation and cell survival. A separate line of evidence has suggested that endostatin-based gene therapy, known for its anti-angiogenic efficacy, may disrupt M2 macrophage function within renal cell carcinoma, contributing to tumor suppression through immune remodeling (82, 90). Taken together, these findings underscore the therapeutic value of TAM modulation in GC and provide a foundation for the development of macrophage-centered treatment strategies to enhance clinical outcomes.

## 6 Conclusion

TAMs represent critical regulators of the GC microenvironment, profoundly shaping immune responses, vascular remodeling, and metastatic behavior. Their phenotypic plasticity enables a dynamic adaptation to tumor-derived signals, often resulting in immunosuppressive and pro-tumor functions. The predominance of M2-like TAMs in GC is closely linked to enhanced epithelial–mesenchymal transition, lymphangiogenesis, immune evasion, and resistance to chemotherapy. The extent and subtype of TAM infiltration carry prognostic significance and may serve as predictive biomarkers for therapeutic responsiveness. Mounting preclinical and translational studies have underscored the therapeutic potential of TAM-targeted interventions, particularly those that inhibit TAM recruitment, suppress M2 polarization, or reprogram existing TAMs toward an antitumoral M1-like state.

Agents such as methionine enkephalin, paclitaxel, and endostatin-based gene therapy exemplify promising tools for TAM modulation. Furthermore, targeting immune checkpoint-expressing TAM subsets or exosome-mediated immunoregulatory circuits may complement existing immunotherapy regimens. Looking forward, future research should prioritize high-resolution dissection of TAM heterogeneity via single-cell RNA sequencing and spatial transcriptomics to reveal distinct functional states and regulatory networks within the tumor microenvironment. Special emphasis should be placed on characterizing and therapeutically targeting immune-suppressive subsets such as PD-1^+^ and LAYN^+^ TAMs, which have demonstrated strong associations with T cell exhaustion and poor prognosis. In parallel, rationally designed combinatorial regimens that integrate TAM-modulating agents with immune checkpoint inhibitors (ICIs) may synergize to overcome immunotherapy resistance and reinvigorate cytotoxic immune responses. These precision strategies hold promise for reshaping the gastric cancer immune landscape and improving durable clinical outcomes.
